# The Outcome Analysis and Complication Rates of Tracheostomy Tube Insertion in Critically Ill Neurosurgical Patients; A Data Mining Study 

**DOI:** 10.29252/beat-070403

**Published:** 2019-10

**Authors:** Veldurti Ananta Kiran Kumar, Narayanam Ananatha Sai Kiran, Valluri.Anil Kumar, Amrita Ghosh, Ranabir Pal, Vishnu Vardhan Reddy, Amit Agrawal

**Affiliations:** 1 *Department of Neurosurgery, Narayna Medical College Hospital, Chinthareddypalem, Nellore-524003, Andhra Pradesh*; 2 *Department of Anesthesia, Narayna Medical College Hospital, Chinthareddypalem, Nellore-524003, Andhra Pradesh*; 3 *Department of Biochemistry, Medical College, 88, College Street, Kolkata-700073*; 4 *Department of Community Medicine, MGM Medical College & LSK Hospital, Kishanganj-855107, Bihar*

**Keywords:** Tracheostomy, Timing, Survival, Outcome

## Abstract

**Objectives:**

To assess the impact, timing, the intra and early post-operative complications and the survival outcome of tracheostomy in critically ill neurosurgery patients.

**Methods:**

This study was a retrospective data mining where data was collected from hospital records from 175 consecutive patients who underwent tracheostomy in the department of Neurosurgery at the Narayna Medical College Hospital, Nellore, India from Jan 2016 to April 2018. A proforma was used to note down the details on the patient status before and after tracheostomy: Glasgow coma scale (GCS), procedure and intra and post-operative complications, type of tracheostomy cannula, details of decannulation, respiration difficulties, and problems with wound, swallowing difficulties, and voice difficulties, stay in intensive care unit (ICU) and hospital and survival status of the patient.

**Results:**

In our series, mean age of TBI cases was 47.42±16.62; mean hospital stay and ICU stay was 18.81±10.22 and 12.58±7.36 days respectively. In all age groups, more tracheostomy was needed in cranial injury cases and surgery was major intervention. Commoner complications were mucous deposition (6.86%), blockage of tracheostomy canula (6.29%), bleeding from multiple attempts (6.06%), excessive bleeding (2.94%). Cranial injury needed tracheostomy more in all age groups and more done at operation theatre without significant improvement of GCS score. Survival was statistically higher after tracheostomy irrespective of GCS status or venue of intervention.

**Conclusion:**

Tracheostomy should be considered as soon as the need for airway access is identified during intervention of the critically ill neurosurgical patients.

## Introduction

Tracheostomy is one of the commonest intensive care unit procedures performed on neurotrauma cases [1, 2].Up to 12% of the 800,000 patients who undergo mechanical ventilation in the United States every year require tracheostomies [3]. Still, tracheostomy has been controversial in the care of traumatic brain Injury (TBI) regarding correlation of optimum timing and desirable outcomes in different dedicated centers. The judgment on tracheostomy in TBI is done on range of clinical decision from the possibility of extubation within two weeks or to cases requiring up to 10 days of mechanical ventilation and even on anticipation of artificial airway for more than 21 days [4]. Other researchers, however, suggested early tracheostomy with severe TBI who have chance of survival with keen watch on the pace of rising Galsgow coma scale with special attention on best motor response [5]. Further, the published literatures do not provide evidence of consistent advantages of tracheostomy across the dissimilar diagnoses [6, 7]. Although tracheostomy is part of the standard operative process in the management of neurotrauma cases, literature search showed paucity of data. The aims and objectives of the current retrospective data analysis was conducted in a tertiary care teaching hospital on to assess the impact of tracheostomy with post-operative complications and the survival outcome in neurosurgery cases during ICU care.

## Materials and Methods

*Study population*

We performed a single centre retrospective data mining of patients where data was collected from hospital records from all the 175 consecutive patients, who underwent tracheostomy in the Intensive care unit (ICU) of the department of Neurosurgery ICU) at a tertiary care dedicated referral trauma center of a teaching hospital. The data mining spread from Jan 2016 to April 2018 at the Narayna Medical College Hospital, Nellore, Andhra Pradesh, India. The protocol was approved by the Institutional Ethics Committee and permission was obtained from concerned authority before the commencement of study. Inclusion criteria: The data on all the patients, without age and gender preference, who underwent tracheostomy in the ICU of department of Neurosurgery were included. Exclusion criteria: The patients who were not admitted in ICU and/or did not undergone tracheostomy in the department of Neurosurgery were excluded from the study.

**Table 1:** Shows diagnosis, management and venue of tracheostomy (n=175).

**Age**	≤20	21-30	31-40	41-50	51-60	61-70	>70	Chi-square (p-value)
	7	28	29	37	27	30	17	
**Diagnosis**								
**Spinal**	0	4	3	10	3	6	2	0.150
**Cranial**	7	24	26	27	24	24	15	
**Management **								
**Surgery**	6	16	22	29	23	24	14	0.370
**Conservative**	1	12	8	8	4	6	3	
**Tracheostomy done at**								
**Bedside**	0	5	5	7	6	4	4	0.919
**Operation theatre**	7	23	24	30	21	26	13	
**Initial assessment of Glasgow Coma Score**								
**≤8**	7	20	23	22	12	13	8	0.078
**>8**	0	8	6	15	15	17	9	
**Before Tracheostomy assessment of Glasgow Coma Score**								
**≤8**	7	22	22	28	20	23	11	0.775
**>8**	0	6	7	9	7	7	6	
**After Tracheostomy assessment of Glasgow Coma Score**								
**≤8**	**6**	**15**	**20**	**27**	**18**	**23**	**10**	0.467
**>8**	**1**	**13**	**9**	**10**	**9**	**7**	**7**	

**Table 2:** ICU stay, Hospital Stay and Survival status in relation to GCS scores after tracheostomy and Venue of tracheostomy in neurosurgery cases

		GCS status after tracheostomy		**Chi square ** **(p-value)**	Venue of tracheostomy		**Chi square (p-value)**
		**GCS ≤8**	**GCS >8**		**Bedside**	**Operation theatre**	
**ICU ** **Stay**	≤3 days (n=7)	5	2	0.589	0	7	0.529
	4-7 days (n=30)	21	9		6	24	
	8-14 days (n=63)	38	25		11	52	
	15-30 days (n-40)	26	14		9	31	
	> 30 days (n=4)	3	1		2	2	
**Hospital stay**	≤3 days (n=3)	2	1	0.142	0	3	0.538
	4-7 days (n=12)	10	2		1	11	
	8-14 days (n=41)	31	10		8	33	
	15-30 days (n=69)	40	29		14	55	
	> 30 days (n=19)	11	8		5	14	
**Survival status**	Alive (n=116)	73	43	0.026	24	92	0.092
	Dead (n=59)	47	12		6	53	
**GCS status after tracheostomy in neurosurgery cases**				GCS ≤8	25	6	0.036
				GCS >8	95	40	

*Study protocol*

A data collection tool was prepared to note down details about the patients’ the socio-demographics viz. age, gender, along with date of admission, date of tracheostomy, date of discharge, contact address and number, diagnosis, initial Glasgow Coma Scale (GCS) on admission and subsequent GCS before and after tracheostomy, procedure and intra and early-OP complications, type of tracheostomy cannula, details of decannulation, ventilator settings before and after the tracheostomy, respiratory difficulties, problems with the wound and associated injuries, swallowing difficulties, and voice difficulties, number of days of ICU stay and hospital stay, and the survival status as well as outcome variables of the patients. Details of the clinical profile during hospital stay and after discharge were noted. The study was conducted by a team which included neuroanesthesia and neurosurgery residents and consultants. The data collection was done by the dedicated co-investigators to ensure consistency of data completeness. Most of the surgeries were performed under local anesthesia, standard tracheostomy technique was followed using either horizontal or vertical incision. Metallic or Portex tracheostomy tubes were used depending on the indication for tracheostomy. The caregivers of the patients were trained in the nursing care of tracheostomy gradually and optimal as well as critical precautions to be undertaken regarding hygiene and feeding. Daily tracheostomy care was provided by the staff nurse, and patients were discharged after regaining adequate consciousness and decannulation of the tracheostomy tube. If patients were discharged with tracheostomy tube, caregivers were adequately trained regarding tracheostomy care along with procurement of suction apparatus and technique of suctioning off the excess pooled secretions before discharge. Patients were followed up till discharge from hospital or death due to any cause during hospital stay and post-discharge, were noted about complications during follow‑up visits and on telephonic conversation up to 6 months.

***Statistical analysis***

Data were entered on Excel and were analyzed using statistical package for social science (SPSS Inc., Chicago, Illinois, USA) for windows, Version 24.0. Armonk, NY: IBM Corp. For categorical variables, frequencies and percentages were determined and Pearson Chi square: Asymptotic significance (2 sided) was used for testing associations and, for continuous variables, descriptive statistics such as mean and standard deviations were computed followed by the *t*-test for comparisons.

## Results

In our case series, mean age of the patients was 47.42 ± 16.62 (ranging from 16 to 64) years. Mean ICU stay was 12.58 ± 7.36 (ranging from2 to 44) days. Mean hospital stay was 18.81 ± 10.22 (ranging from 3to 53) days. Majority had cranial injury in comparison to spinal injury needing tracheostomy in all the age groups; yet this difference was not significant. Surgery was major intervention; though conservative management was also practiced in all age groups; yet this difference was not significant. Tracheostomy was done at operation theatre in majority of time, though bedside procedure was done sometimes in emergency situations; this difference was also not significant. Initial assessment of Glasgow Coma Score and assessment before tracheostomy did not have significant effect after tracheostomy in our study (Table 1). 

Majority of tracheostomy in neurosurgical cases with GCS less than and more than 8 after tracheostomy stayed in ICU 8-14 days followed by 15-30 days. On the contrary, majority of tracheostomy cases with GCS less than and more than 8 after tracheostomy stayed in Hospital 15-30 days followed by 8-14 days. Survival was statistically higher after tracheostomy whether GCS less than and more than 8. Majority of tracheostomy in neurosurgical cases was done in Bedside and Operation theatre stayed in ICU 8-14 days followed by 15-30 days. On the contrary, majority of tracheostomy cases with done in Bedside and Operation theatre stayed in Hospital 15-30 days followed by 8-14 days. Survival was not statistically significant after tracheostomy whether tracheostomy was done in Bedside and Operation theatre. Tracheostomy cases with GCS less than and more than 8 after tracheostomy compared to tracheostomy done in Bedside and Operation theatre found statistically significant (Table 2).

Common complications following tracheostomy were mucous deposition (6.86%), frequent blockage of cannula (6.29%), bleeding from multiple attempts (6.06%), excessive bleeding (2.94%), blood clots (1.71%), paratracheal insertion (1.16%), posterior tracheal wall laceration (0.57%), and pneumothorax (0.57%).

## Discussion

Tracheostomy helps in airway management and lessens ventilator‑associated pneumonia. Patients may have shorter intensive care unit stays, days of mechanical ventilation, and hospital stays. As soon as the need for prolonged airway access is identified, the tracheostomy should be considered [1, 2]. Normally, this decision is undertaken by second week of in-patient’s care and bedside techniques permit prompt tracheostomy with lower morbidities. Tracheostomy is commonly done procedure, yet the complications remain high. The goal of this study was to evaluate the impact, timing, the intra and early post-operative complications and the survival outcome of tracheostomy in Neurosurgery patients (ICU). The decision to do tracheostomy depended on type of injury where cranial injury needed tracheostomy more in all age groups and was done at operation theatre in majority of time, without significant improvement of GCS score after tracheostomy in neurosurgical cases.

Further, the advantages of tracheostomy include relief of respiratory distress, opportunity for oral hygiene and oral feeding, and safer as well as easier nursing care for airway than translaryngeal intubation [1, 2]. In our study, tracheostomy after neurosurgical interventions with GCS less than and more than 8 after tracheostomy compared to tracheostomy done in Bedside and Operation theatre found statistically significant. Tracheostomy did not have any significant effects in ICU or Hospital stay though Survival was statistically higher after tracheostomy with GCS less than and more than 8, yet not whether tracheostomy was done in Bedside and Operation theatre. Research groups reported early tracheostomy reduced number of ICU days though in critically ill patients, death would not be caused by tracheostomy alone. Mortality rate in ICU reduced in early tracheostomy cases contrary to some studies [8].Other published literatures also reported the same observations on tracheostomy [9].

In a retrospective study of large sample size, results showed increased mortality rate in late tracheostomy cases (>10 days) followed by intermediate tracheostomy [5–9 days] followed by early tracheostomy (<4 days) [10]. In an Indian study, the researchers practiced an innovative cost-effective way of long term follow up care of the tracheostomy cases. In cases of excessive secretions in very severe head injury, when it took prolonged decannulation and hospital stay with financial constraints of patients, the domiciliary caregivers were provided adequate training in tracheostomy as well as nursing care. Further, they were asked to procure a small foot operated or electric suction apparatus with capacity building in sucking off the excess pooled secretions before the patient were discharged [8]. An Egyptian study reported that there were significantly longer durations of ICU stay in tracheostomized patients [11].

ICU length of stay and mean predicted mortalityfor tracheostomized patients who had the tracheostomy after 10 days was significantly higher than in those done within 10 days [12]. Literature reported reduced direct variable and likely total hospital costs in ICU length of stay with additional benefits of early tracheostomy in in reducing hospital stays [3, 13]. Often after tracheostomy patients leave ICU breaking continuity of care with the neuro-surgical emergency team members who seldom get information on follow-up of these patients. Further, clinical practice guidelines often vary from data of well controlled clinical trials with huge heterogeneity in the reported mortality in randomized studies. 

***Complications***

There are risks in and acute stages and on long-term follow-up for which decision regarding tracheostomy must be individually considered as the need for prolonged airway access is identified which is generally made within 7–10 days. Bedside techniques allow rapid tracheostomy with low morbidity, shorter days of intensive care, mechanical ventilation, and hospital stays. Researchers have noted that the efficiency of surgical teams conducting tracheostomy and supportive hospital services with standardized operative procedures as well as hospital-based protocols for tracheostomy insertion and care has been associated with improved outcomes. The clinical studies on late complications of tracheostomy once the patient gets discharged from the hospital are lacking. Most of the patients undergo decannulation of tracheostomy tube before discharge, but few patients with excessive secretions and those who sustained very severe head injury need long‑term care before (and after as well) decannulation. Research groups have reported benefits of early tracheostomy in neurotrauma cases with severely impaired consciousness and shorter ICU and hospital stay compared to late tracheostomy with no significant effect on mortality [14, 15].

Reviewers in this field of research opined that the list of complications may appear formidable, but this should not prevent the surgeon from performing a tracheostomy in a patient who clearly stands to benefit from one. Bleeding remains one of the most common intraoperative complications during a tracheostomy, although major hemorrhage remains rare [16]. In a case series of 100 cases of tracheostomy performed by the researchers, a complication rate of 48% was observed; no death was reported; the complication rate following emergency tracheostomy was twice than elective approach; younger patients had higher complications. The research group felt that to reduce complications is based on how to convert an emergency situation of acute airway obstruction to elective ones. Obstruction of tracheostomy tube was a common complication [17]. The Egyptian study found no significant early complications after tracheostomy, but still laryngeotracheal stenosis was important reported late complications; early tube obstructions were reported among 5.6% [11].

In our study, common complications following tracheostomy were mucous deposition (6.86%), frequent blockage of cannula (6.29%), Bleeding from multiple attempts (6.06%), Excessive bleeding (2.94%). Researchers from other centers reported short-term complication like pneumothorax, damage to trachea and other adjacent organs, bleeding, and infections, and long-term complications may arise relating to long‑standing artificial airway [18]. A systemic review reported by Siempos *et al*. also mentioned that early or late tracheostomy did not show much difference in terms of complications [9]. Early tracheostomy can reduce respiratory problems such as ventilator-associated pneumonia and sepsis [9, 10]. Late tracheostomy cases can have more complications – bleeding, stoma infections, granuloma, and tracheal stenosis [19].

***Strengths*** ***and limitations***

The strength of this study is that to the horizon of our knowledge, this was a pioneering study on assess impact of age-group and GCS in the intra and early post-operative complications with overall survival outcomes of tracheostomy in Neurosurgery patients (neuro-ICU). So, firstly, we had done exhaustive analysis of available data to find out factors of optimum outcomes with minimum complication of tracheostomy in neuro-ICU. Further, this study demonstrated that heterogeneity in the patients of neurosurgery is a hurdle to improve outcomes after tracheostomy. Our study suggested that though, majority of neurotrauma patients require tracheostomy for long term ventilator support and associated speech and swallowing problems are expected.

We had several limitations. Firstly, this was aretrospective data mining where variable and parameters were decided earlier. Also it is not clear if early tracheostomy reduced risk of relevant complications such as aspiration, pneumonia, tracheal stenosis etc. Secondly, Information on type of underlying CNS disorder led to ICU admission was missing as well as more detailed surgical or medical treatment. Thirdly, in a retrospective data mining there was potential limitation of missing data and inability to cross-check quality of data entry. Lastly, with best of our effort, we are yet to find sufficient numbers of relevant published literatures or national-level discourse regarding our research question from Indian subcontinent.

In conclusion, tracheostomy should be done as the need for airway access is identified during intervention. The finding of this study can be extrapolated to improve the tracheostomy related health issues and safety to the patients for better outcome. In the next phase of our study we will explore all the correlates of the complications of tracheostomy with special attention to their management and prevention to understand deeply with an opportunity to render a prospective study regarding need, methods and prognosis after tracheostomy of neurosurgical intervention.

## Financial support and sponsorship: 

**None.**

## Conflicts of interest:

**There are no conflicts of interest.**

## Ethical conduct of research:

**This study followed ICMR guidelines regarding research on human participants.**
